# Prevalence of alcohol use over time in survivors of head and neck cancer

**DOI:** 10.1016/j.amjoto.2025.104743

**Published:** 2025-10-20

**Authors:** M. Bryant Howren, Alan J. Christensen, Nitin A. Pagedar

**Affiliations:** a Center for Access & Delivery Research and Evaluation (CADRE), VA Iowa City Health Care System, Iowa City, IA, United States of America; b Department of Internal Medicine, Carver College of Medicine, University of Iowa, Iowa City, IA, United States of America; c Holden Comprehensive Cancer Center, University of Iowa, Iowa City, IA, United States of America; d Department of Psychology, East Carolina University, Greenville, NC, United States of America; e Department of Otolaryngology—Head and Neck Surgery, Carver College of Medicine, University of Iowa, Iowa City, IA, United States of America

**Keywords:** Alcohol use, Cancer survivorship, Head and neck cancer, Health-related quality of life, Psycho-oncology

## Abstract

**Objectives:**

Alcohol use is a risk factor for the development of head and neck cancer (HNC) and continued use after diagnosis is associated with recurrence, comorbidities, and poor psychosocial outcomes. This retrospective observational descriptive study sought to report the prevalence of alcohol use in survivors of HNC from diagnosis through 10 years postdiagnosis.

**Methods:**

Adult patients with upper aerodigestive tract carcinomas from the head and neck oncology clinic at a large Midwestern healthcare system were eligible to participate. Between 1998 and 2014, 2095 patients reported alcohol use status at diagnosis. By 10 years postdiagnosis, this number was 187. Self-reported alcohol use was classified as current/previous/never.

**Results:**

At diagnosis, 48.8 % reported currently using alcohol, 36.6 % reported previous use, and 14.7 % reported never using; 24.5 % of those with a history of alcohol use were likely problem users. At 1 year postdiagnosis, 44.7 % reported currently using alcohol, 40.2 % reported previous use, and 15.1 % reported never using. At 5 years postdiagnosis, 48.9 % reported currently using alcohol, 38.3 % reported previous use, and 12.8 % reported never using. At 10 years postdiagnosis, 42.2 % reported currently using alcohol, 44.9 % reported previous use, and 12.8 % reported never using alcohol.

**Conclusions:**

Continued alcohol use remains an issue over time in HNC survivors. Screening protocols should be incorporated into clinical workflow and more research is needed to understand correlates and degree of use in long-term HNC survivors to facilitate optimal recovery and adjustment during the survivorship period.

## 1. Introduction

Alcohol use is a known risk factor for the development of head and neck cancer (HNC) and continued use after diagnosis is associated with cancer recurrence, significant comorbidities, and poor psychosocial outcomes including health-related quality of life (HRQOL) [1–4]. Research indicates that between 30 to 60 % of HNC patients continue alcohol use after diagnosis, with many continuing to exhibit this behavior well into the survivorship period [1,5,6]. The present retrospective observational descriptive study sought to add to existing evidence of alcohol use in patients with HNC by reporting its prevalence across time in a large sample of patients including long-term survivors up to 10 years postdiagnosis.

## 2. Methods

### 2.1. Participants & procedure

Adult patients with upper aerodigestive tract carcinomas from the head and neck oncology clinic at the University of Iowa Hospitals and Clinics were eligible to participate in the Outcomes Assessment Project (OAP), designed as a prospective longitudinal observational study of outcomes in HNC. At diagnosis, patients were approached regarding participation and consented in writing if interested. The OAP parent study successfully recruited approximately 75 % of all eligible patients with HNC seen from November 1998 through December 2014. Clinical and psychosocial data was obtained via self-report or the medical record including site and stage of cancer, treatment modality, survival outcome, demographics, and other clinical and psychosocial characteristics, beginning at study enrollment to coincide with the time of diagnosis. Based on healthcare team recommendations, patients were seen at three-month intervals after diagnosis during the first year of treatment and follow-up; after 1 year, research assessments occurred annually through the fifth year postdiagnosis, then again at the tenth year postdiagnosis. All procedures were approved by the University of Iowa’s IRB (#199412746). The present study examines alcohol use retrospectively in the total sample enrolled beginning in 1998.

### 2.2. Measurement of key variables

#### 2.2.1. Alcohol use

Patients reported alcohol consumption at each time point as being current, previous, or never. Problem alcohol use, collected only at diagnosis, was captured using the Short Michigan Alcoholism Screening Test (SMAST), a self-report screening tool designed to detect problem drinking and alcohol use disorder (AUD); adequate reliability and validity have been reported [7]. Items are presented in yes/no format, with scores ranging from 0 to 13. A score of 2 suggests possible alcohol abuse and a score of 3 or higher suggests probable alcohol abuse [7]. The SMAST was added to the study protocol during a subsequent iteration after parent study initiation—and thus is not available for all patients—and collected only for those indicating alcohol use. It is presented here to indicate the number at diagnosis who may have exhibited problem intake.

## 3. Results

Table 1 details demographic and clinical characteristics of the sample at diagnosis (*N* = 2095). Most patients were White (95.8 %), married (64.9 %), and presented with advanced stage disease (56.2 %). Table 2 shows rates of alcohol use in survivors; rates of use are presented for diagnosis, 1 through 5 years postdiagnosis, and 10 years postdiagnosis. Use remained consistent across time with greater than 40 % of patients indicating current use at each follow-up time point. Specifically, at diagnosis, 48.8 % reported currently using alcohol, 36.6 % reported previous use, and 14.7 % reported never using. Of the 2095 with data at diagnosis, 1327 were administered the SMAST with 24.5 % scoring 3+ (data previously reported; see [8]), which is suggestive of problem use. At 1 year postdiagnosis, 44.7 % reported currently using alcohol, 40.2 % reported previous use, and 15.1 % reported never using. At 5 years postdiagnosis, 48.9 % reported currently using alcohol, 38.3 % reported previous use, and 12.8 % reported never using. At 10 years postdiagnosis, 42.2 % reported currently using alcohol, 44.9 % reported previous use, and 12.8 % reported never using. Rates for all years captured are reported in Table 2, which shows a very consistent pattern of continued use over time.

## 4. Discussion

The present study further supports that continued alcohol use is an issue across time postdiagnosis in HNC survivors, including those who have survived long-term. Rates of continued use remained remarkably stable and high in this sample, which aligns with other studies reporting continued alcohol use in HNC [1,5,6,9]. For example, in a narrative review of studies, Miller et al. reported prevalence rates of continued use between 34 % and 57 % postdiagnosis [5]. Additionally, Teckie and colleagues reported that while there was a decrease in use in the immediate months after HNC treatment, the degree of use increased over time and surpassed baseline levels by 24 months [6]. These results indicate that more research is needed into factors associated with continued use, including problematic use. A position paper by the American Society of Clinical Oncology specifically advocates for increased research into the identification of cancer patients who are using alcohol or may be at risk for relapse [10]. McCarter and colleagues also note that there has been considerably less research regarding alcohol use as compared to other behavioral risk factors such as tobacco use in HNC patients [11]; given that estimates indicate approximately 75 % of HNC is connected to tobacco and/or alcohol, increased research is warranted [12].

Although this study cannot differentiate between problem use and occasional use, previous studies—including those part of this observational parent study—have identified approximately one quarter of patients as having problem use at diagnosis and research suggests a dose-response relationship between alcohol use and HNC development [1,8]. Patients may struggle to reduce risky or problematic use or consume alcohol as a means of coping [13,14]. These results also suggest that all patients with HNC be screened for problem alcohol use at diagnosis and counseled regarding the potentially harmful effects of continued drinking [10]. There are several brief screening tools that can be readily incorporated into the clinic environment to assess the need for more in-depth evaluation, such as the widely used brief version of the Alcohol Use Disorders Identification Test (AUDIT-C). For those with excessive alcohol use, brief interventions may assist in reducing binge drinking and high weekly intake [15], or referral to substance use treatment services may be required for highly problematic cases.

This study is not without limitations. Most respondents were White and from only one Midwestern healthcare system, necessitating further study given the changing demographics of HNC. We also do not have information about degree or frequency of consumption other than a baseline measure of problem use, so are unable to differentiate those with problematic use from occasional use over time, which has implications for treatment efficacy, complications, and HRQOL. However, the rates reported here align with other studies of alcohol use in HNC, but more detailed studies to examine the presence, degree, and clinical relevance of continued use over time are needed. Available information at diagnosis also suggests that, of those who indicated current drinking, a quarter exhibited problem use. We also have no information regarding patient HPV status in this sample, suggesting additional research is needed to examine how alcohol use varies with HPV-associated disease. Lastly, this study—despite known limitations—was undertaken to provide descriptive data to underscore the need for additional work regarding alcohol use in the HNC population. While conclusions may be limited, it is nonetheless concerning for researchers and clinicians that alcohol continues to be an issue for many patients well into the survivorship period even though much evidence indicates negative outcomes associated with continued use in treatment and recovery contexts. Others have also advocated that there is a need to give alcohol use more attention in HNC, aligning it with attention given other behaviors such as tobacco use.

## 5. Conclusion

The present study indicates that continued alcohol use after diagnosis in HNC remains an issue over time and provides valuable prevalence estimates for an understudied behavioral risk factor in HNC. Screening protocols for alcohol use should be implemented into clinical workflow beginning at diagnosis with consideration of, or referral to, alcohol risk reduction interventions as necessary to ensure needs over the survivorship care trajectory are met for patients with HNC.

## Figures and Tables

**Table 1:** Patient, disease, and treatment characteristics at diagnosis (*N* = 2095).

Age	
Mean (SD)	60.3 (12.6)
Marital status	
Married/living with partner	1311 (64.9 %)
Unmarried/divorced/widowed	784 (35.1 %)
Race	
White	1956 (95.8 %)
Black/Other	139 (4.2 %)
Sex	
Male	1430 (68.3 %)
Female	665 (31.7 %)
Site	
Oral cavity	715 (34.1 %)
Oropharynx	448 (21.4 %)
Hypopharynx	79 (3.8 %)
Larynx	481 (23.0 %)
Else/unknown	372 (17.8 %)
Stage	
Early (0–2)	726 (34.7 %)
Advanced (3–4)	1178 (56.2 %)
Not stageable/unknown	191 (9.1 %)
Treatment	
Surgery only	687 (32.8 %)
Radiotherapy only	281 (13.4 %)
Combination (w/ or w/out chemo)	898 (42.9 %)
Other/unknown	229 (10.9 %)
Tobacco use	
Current	780 (37.2 %)
Previous	890 (42.5 %)
Never	415 (19.8 %)
Unknown	10 (<1 %)

**Table 2:** Prevalence of alcohol use at diagnosis through 10 years postdiagnosis.

	Diagnosis N = 2095	1 yr postdx *N* = 1199	2 yr postdx *N* = 568	3 yr postdx *N* = 415	4 yr postdx *N* = 329	5 yr postdx *N* = 397	10 yr postdx *N* = 187
Alcohol Use							
Current	1022 (48.8 %)	536 (44.7 %)	295 (51.9 %)	208 (50.1 %)	162 (49.2 %)	194 (48.9 %)	79 (42.2 %)
Previous	766 (36.6 %)	482 (40.2 %)	209 (36.8 %)	154 (37.1 %)	121 (36.7 %)	152 (38.3 %)	84 (44.9 %)
Never	307 (14.7 %)	181 (15.1 %)	64 (11.3 %)	53 (12.7 %)	46 (14.0 %)	51 (12.8 %)	24 (12.8 %)

## Data Availability

The data that support the findings of this article are available from the corresponding author upon request.
